# Reduced Cardiovascular Mortality 10 Years after Supplementation with Selenium and Coenzyme Q10 for Four Years: Follow-Up Results of a Prospective Randomized Double-Blind Placebo-Controlled Trial in Elderly Citizens

**DOI:** 10.1371/journal.pone.0141641

**Published:** 2015-12-01

**Authors:** Urban Alehagen, Jan Aaseth, Peter Johansson

**Affiliations:** 1 Division of Cardiovascular Medicine, Department of Medical and Health Sciences, Linköping University, Linköping, Sweden; 2 Research Department, Innlandet Hospital Trust and Hedmark University College, Elverum, Norway; Maastricht University, NETHERLANDS

## Abstract

**Background:**

Selenium and coenzyme Q10 are important antioxidants in the body. As the intake of selenium is low in Europe, and the endogenous production of coenzyme Q10 decreases as age increases, an intervention trial using selenium and coenzyme Q10 for four years was performed. As previously reported, the intervention was accompanied by reduced cardiovascular mortality. The objective of the present study was to analyze cardiovascular mortality for up to 10 years after intervention, to evaluate if mortality differed in subgroups differentiated by gender, diabetes, ischemic heart disease (IHD), and functional class.

**Methods:**

Four-hundred forty-three healthy elderly individuals were included from a rural municipality in Sweden. All cardiovascular mortality was registered, and no participant was lost to the follow-up. Based on death certificates and autopsy results mortality was registered.

**Findings:**

Significantly reduced cardiovascular mortality could be seen in those on selenium and coenzyme Q10 intervention. A multivariate Cox regression analysis demonstrated a reduced cardiovascular mortality risk in the active treatment group (HR: 0.51; 95%CI 0.36–0.74; P = 0.0003). The reduced mortality could be seen to persist during the 10-year period. Subgroup analysis showed positive effects in both genders. An equally positive risk reduction could be seen in those with ischemic heart disease (HR: 0.51; 95%CI 0.27–0.97; *P* = 0.04), but also in the different functional classes.

**Conclusions:**

In a 10-year follow-up of a group of healthy elderly participants given four years of intervention with selenium and coenzyme Q10, significantly reduced cardiovascular mortality was observed. The protective action was not confined to the intervention period, but persisted during the follow-up period. The mechanism explaining the persistency remains to be elucidated. Since this was a small study, the observations should be regarded as hypothesis-generating.

## Introduction

The trace element selenium is essential for all living cells[1, 2]. It is mostly found as selenoproteins in the body, including glutathione peroxidases, thioredoxin reductase and selenoprotein P, which protect against oxidative stress. Increased vascular oxidative stress and endothelial dysfunction in patients with coronary heart disease have been reported[3, 4]. However, the association between ischemic heart disease and suboptimal selenium intake has been a controversial topic and there are conflicting results regarding the effectiveness of selenium intervention in patients with ischemic heart disease[5–8]. As the selenium content in the soil is low in Europe compared to the US, supplementation or biofortification has been regarded as logical in European regions[9, 10]. The mean intake of selenium in Europe is estimated to be about 40 μg/day, whereas the estimated mean intake in the US is 134 μg/day in males and 93 μg/day in females. These inter-continental differences in selenium intake provide an explanation for inconsistent results of selenium supplementation that are reported in one Cochrane review[11]. However, there are some reports from small study samples in Europe indicating a higher mortality in those with low selenium intake[12–14]. Our research group have recently reported higher cardiovascular mortality in a community population with low plasma selenium concentration[15].

Xia et al. demonstrated an interrelationship between selenium and coenzyme Q10 (ubiquinone) in the metabolic pathway to the active form of coenzyme Q10 (ubiquinol). Moreover, an adequate presence of coenzyme Q10 is needed for optimal synthesis of selenocysteine-containing enzymes[16]. Similarly, a deficiency of selenium could influence the ability to get adequate concentrations of active coenzyme Q10 in cellular compartments. Coenzyme Q10 has been shown to have effects on the endothelial function[17]. It is also known that coenzyme Q10 is a powerful anti-oxidant, mainly against lipid peroxidation[18]. Reports have shown that ubquinone also reduces inflammatory response [19] in those with diabetes [20]. It is reported that the endogenous production of coenzyme Q10 decreases after the age of 20, and the myocardial production is reduced to half at the age of 80 [21]. Thus, elderly people living in geographical areas with low selenium content in the soil and in their food may be at increased risk of heart disease and premature death.

A dietary supplementation trial with both selenium and coenzyme Q10 to 443 elderly Swedish community members was performed during 2003 until 2010. The main results have previously been published[22]. The intervention time was four years, and the median follow-up time was 5.2 years. The main results were significantly reduced cardiovascular mortality, improved cardiac function as evaluated on echocardiography, and a reduced increase of the N-terminal fragment of proBNP (NT-proBNP), a cardiac peptide biomarker. Recently, we also have reported increased health-related quality of life in those with active treatment, but also data showing more days out of hospital in this elderly population (in press). However, less is known about possible long-term effects of the supplementation in the post-intervention period. Also, it would be of interest to separately evaluate specific sub-groups such as participants with diabetes, those with a history of IHD, and those in different functional classes, to see if specific sub-groups benefit more or less from this intervention.

The primary aim of the present study was to evaluate the cardiovascular effects of the intervention in the population under study 10 years after the introduction of a four-year period of supplementation. A secondary aim was to establish if there were different effects on CV-morality in gender, diabetes, IHD and functional classes as measured by New York Heart Association functional Class (NYHA class).

## Materials and Methods

The design of the main study has been published elsewhere[22]. In brief, 443 elderly healthy participants were given dietary supplementation of 200 mg/day of coenzyme Q10 capsules (Bio-Quinon 100 mg B.I.D, Pharma Nord, Vejle, Denmark) and 200 μg/day of organic selenium yeast tablets (SelenoPrecise 200 μg, Pharma Nord, Vejle, Denmark), or a similar placebo. The study supplementation was taken in addition to regular medication. All study medication (active drug and placebo) not consumed were returned and counted. All participants were examined by one of three experienced cardiologists. A new clinical history was recorded, and a clinical examination was performed, including blood pressure, assessment of New York Heart Association functional class (NYHA class) as well as ECG and echocardiography. Doppler echocardiographical examinations were performed with the participant in the left lateral position. The ejection fraction (EF) readings were categorized into four classes with interclass limits placed at 30%, 40% and 50% [23, 24]. Normal systolic function was defined as EF≥ 50%, while severely impaired systolic function was defined as EF*<* 30%.

As the intervention time was unusually long, 48 months, only 228 participants completed the study, 86 died during the total intervention time, and 129 (29.1%) decided not to complete the study. The reasons for the latter have been presented in detail in the main publication, but the main reason was too many tablets to take [22]. Written, informed consent was obtained from all patients. The study was approved by the Regional Ethical Committee in Linköping, Sweden, and conforms to the ethical guidelines of the 1975 Declaration of Helsinki. This study was registered at Clinicaltrials.gov, and has the identifier NCT01443780

### Biochemical analyses

All blood samples were obtained while the patients were at rest in a supine position. The blood samples were collected in plastic vials containing EDTA (ethylenediamine tetracetic acid). The vials were placed on ice before chilled centrifugation at 3000g, and then frozen at -70°C. No sample was thawed more than twice. NT-proBNP 1–76 was measured on the Elecsys 2010 platform (Roche Diagnostics, Mannheim, Germany). The total coefficient of variation was 4.8% at 26 pmol/L and 2.1% at 503 pmol/L (n = 70).

### Mortality

All instances of cardiovascular mortality (CV mortality) were registered. The mortality information was obtained from the National Board of Health and Welfare in Sweden, which registers all deaths of Swedish citizens based on death certificates or autopsy reports. Written, informed consent was obtained from all patients.

### Statistical methods

Descriptive data are presented as percentages or mean ± SD. A Student’s unpaired two-sided *T*-test was used for continuous variables and the Chi-square test was used for discrete variables. Kaplan-Meier analysis was used to demonstrate CV mortality during the follow-up period. Cox proportional hazard regression analysis was used to evaluate risk of cardiovascular mortality. The independent variables included in the multivariate model were variables known to be associated with CV-mortality: age, smoking, hypertension, diabetes, ischemic heart disease, New York Heart Association functional (NYHA) class III, Hb<120g/L, EF<40% and 4^th^ quartile of NT-proBNP.

A P-value of <0.05 was considered statistically significant. All data were analyzed using standard software (Statistica v. 12.5, Statsoft Inc, Tulsa, OK).

## Results

The study population were followed regarding CV mortality during a median follow-up time of 3668 days (range 348–4488), thus 10 years. In the survivor group the median follow-up time was 3836 days (range 689–4488), and in the non-survivor group a median follow-up time of 2505 days (range 348–4347) was recorded.

From the basal characteristics it could be seen that the two populations were balanced at the start of the intervention in all but one variable, use of ACE-inhibitors (15.8% vs 24.3%; *P* = 0.03)(Table 1).

**Table 1:** Population characteristics at inclusion, at end of the intervention, and after 10 years.

	At study start			At the end of intervention			After 10 years		
	Active	p-value	Placebo	Active	p-value	Placebo	Active	p-value	Placebo
n	221		222	208		194	175		136
Age, mean	78		78	82		82	88		88
Males/Females, n	115/106		110/112	105/103		90/104	81/94		61/75
Smokers, n (%)	21 (9.5)	0.86	20 (9.0)	17 (8.2)	0.98	16 (8.2)	10 (5.7)	0.74	9 (6.6)
Diabetes, n (%)	47 (21.3)	0.93	48 (21.6)	44 (21.2)	0.99	41 (21.1)	33 (18.9)	0.54	22 (16.2)
Hypertension, n (%)	158 (71.5)	0.32	168 (75.7)	148 (71.2)	0.30	147 (75.8)	123 (70.3)	0.44	101 (74.3)
IHD, n (%)	47 (21.3)	0.51	53 (23.9)	44 (21.2)	0.81	43 (22.2)	28 (16.0)	0.97	22 (16.2)
NYHA class I, n (%)	118 (53.4)	0.32	108 (48.6)	115 (55.3)	0.34	98 (50.5)	103 (58.9)	0.70	83 (61.0)
NYHA class II, n (%)	61 (27.6)	0.77	64 (28.8)	56 (26.9)	0.58	57 (29.4)	47 (26.9)	0.83	38 (27.9)
NYHA class III, n (%)	41 (18.6)	0.49	47 (21.2)	36 (17.3)	0.74	36 (18.6)	25 (14.3)	0.21	13 (9.6)
NYHA class IV, n (%)	0		0	0		0	0		0
Unclassified, n	1		3	1		3	0		2
Medical Treatment									
ACEI, n (%)	35 (15.8)	0.03	54 (24.3)	33 (15.9)	0.04	47 (24.2)	23 (13.1)	0.11	27 (19.9)
Beta blockers, n (%)	81 (36.7)	0.40	73 (32.9)	76 (36.5)	0.46	64 (33.0)	62 (35.4)	0.26	40 (29.4)
Digitalis, n (%)	11 (5.0)	0.99	11 (5.0)	10 (4.8)	0.94	9 (4.6)	7 (4.0)	0.86	6 (4.4)
Diuretics, n (%)	70 (31.7)	0.08	88 (39.6)	64 (30.8)	0.06	77 (39.7)	52 (29.7)	0.52	45 (33.1)
Statins, n (%)	45 (20.7)	0.50	51 (23.0)	42 (20.2)	0.46	45 (23.2)	36 (20.6)	0.87	29 (21.3)
Examinations									
Hb<120g/L, n (%)	23 (10.4)	0.39	29 (13.1)	21 (10.1)	0.47	24 (12.4)	17 (9.7)	0.96	13 (9.6)
EF<40%, n (%)	16 (7.2)	0.87	17 (7.7)	15 (7.2)	0.68	12 (6.2)	10 (5.7)	0.41	5 (3.7)

Note: ACEI: ACE- inhibitors; EF: Ejection fraction according to echocardiography; IHD: Ischemic heart disease; NYHA: New York Heart Association functional class

At the start of the intervention the two groups were the same age (78 years), the relation between males/ females was well balanced, and the proportion of smokers was equal between the two groups (9%). Of the total population, 21% had diabetes, which was the same in the two groups, and the proportions with hypertension were equally well balanced (71.5% vs. 75.7%). The proportions suffering from IHD were equal in the two groups (21.3% vs. 23.9%). The clinical characteristics of the two groups active treatment and placebo are also demonstrated at end of the intervention, and after 10 years of follow-up (Table 1). It could be seen that the part of participants classified to NYHA class III, that is those with a marked limitation of physical activity, is more reduced in the placebo group after 10 years, compared to start of intervention, or at the stop of the intervention, and compared to the active treatment group. The same trend could be seen regarding the part with at least moderately reduced systolic cardiac function (EF<40%) in the placebo group (Table 1).

### Cardiovascular mortality within 10 years

**Fig 1:**
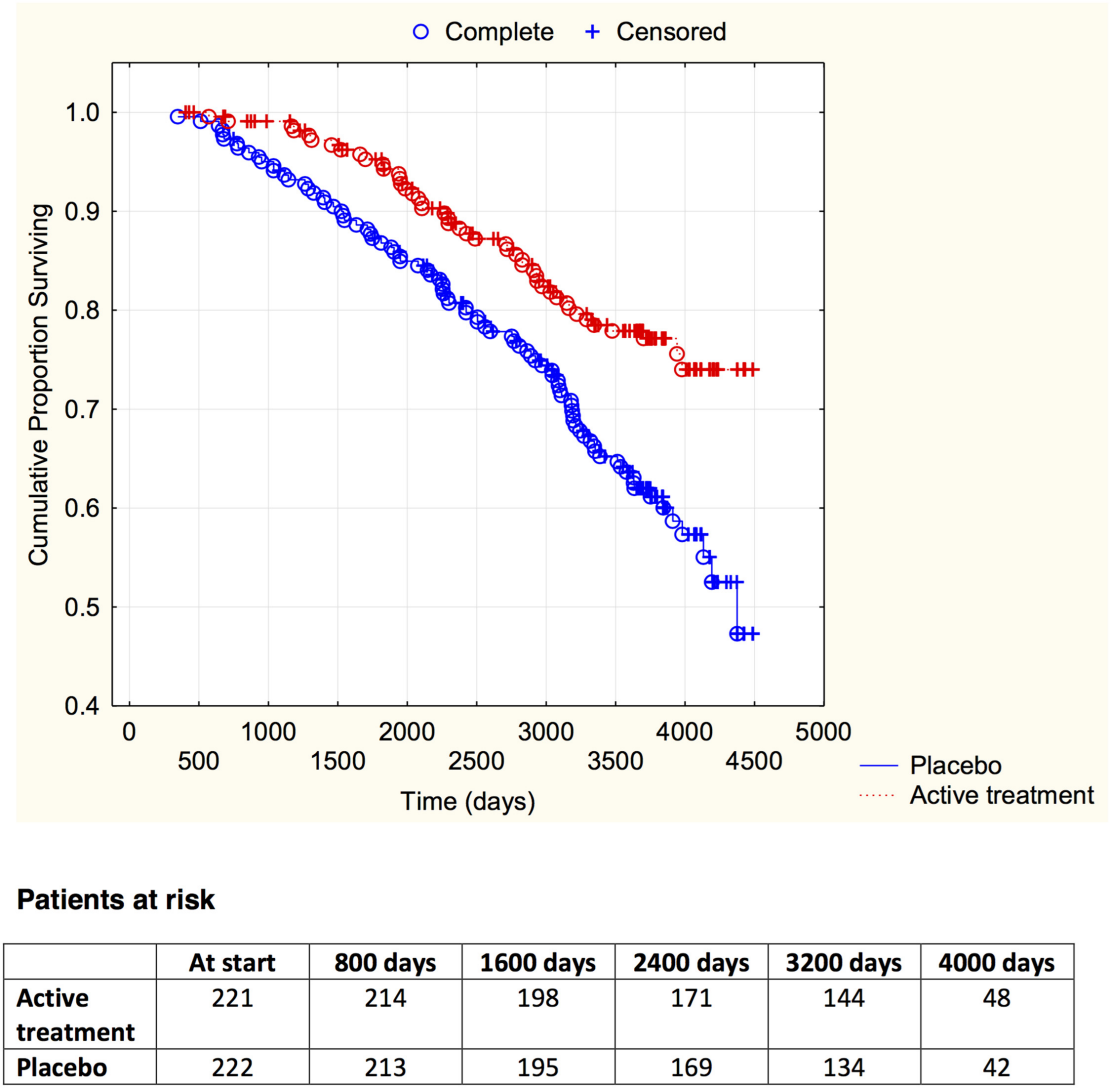
Kaplan-Meier graph illustrating cardiovascular mortality in the study population in those supplemented with selenium and coenzyme Q10 versus placebo during four years on top of regular pharmaceutic treatment during a follow-up period of 10 years.

Evaluating the mortality using a follow-up time of 10 years revealed a lower CV mortality in the active treatment group compared to the placebo group (46/221 vs. 86/222; *χ* ^2^:17.01; *P* = <0.0001). The CV mortality during the follow-up period is demonstrated in a Kaplan-Meier graph, where the two groups are indicated (Fig 1).

Evaluating risk, the active treatment group displayed significantly reduced cardiovascular risk compared to the placebo group in the univariate Cox regression analysis (HR: 0.52; 95%CI 0.36–0.74; *P* = 0.0004). If applying a multivariate model including well-known variables influencing cardiovascular risk, and the well-known biomarker NT-proBNP to the model, a significant risk reduction could be displayed by the active supplementation of selenium and coenzyme Q10 (HR: 0.51; 95%CI 0.36–0.74; P = 0.0003) (Table 2).

**Table 2:** Cox proportional hazard regression analysis evaluating risk of cardiovascular mortality by supplementation of selenium and coenzyme Q10 combined in a multivariate model after 10 years of follow-up after 4 years of intervention to an elderly community population.

Variables	Hazard ratio	95%CI	*P*-value
Age	1.12	1.06–1.18	<0.0001
Smoking	2.04	1.26–3.29	0.004
Hypertension	1.32	0.87–2.01	0.19
Diabetes	1.59	1.09–2.32	0.02
IHD	1.38	0.92–2.06	0.12
NYHA class 3	2.05	1.38–3.06	0.0004
Hb<120g/L	0.95	0.59–1.52	0.82
EF<40%	0.75	0.42–1.33	0.33
NT-proBNP Q4	2.26	1.52–3.38	<0.0001
Active treatment	0.51	0.36–0.74	0.0003

Notes: EF: Ejection fraction; IHD: Ischemic heart disease; NT-proBNP: N-terminal fragment of proBNP; NYHA: New York Heart Association functional class; Q4: 4^th^ quartile

### Subgroup analyses

Dividing the study population into the two genders revealed significantly reduced cardiovascular mortality in both genders (males active treatment 34/115 vs. 49/110; *χ* ^2^:5.42; *P* = 0.02; females active treatment 12/106 vs. 37/112; *χ* ^2^:8.31; *P* = 0.004).

Evaluating those with a history of diabetes showed a reduced CV mortality for those on active treatment (14/47 vs. 26/48; *χ* ^2^:5.79; *P* = 0.0016). Applying these data into a univariate Cox proportional hazard regression analysis showed reduced cardiovascular risk for those on active treatment (HR: 0:51; 05%CI 0.27–0.98;*P* = 0.04).

**Fig 2:**
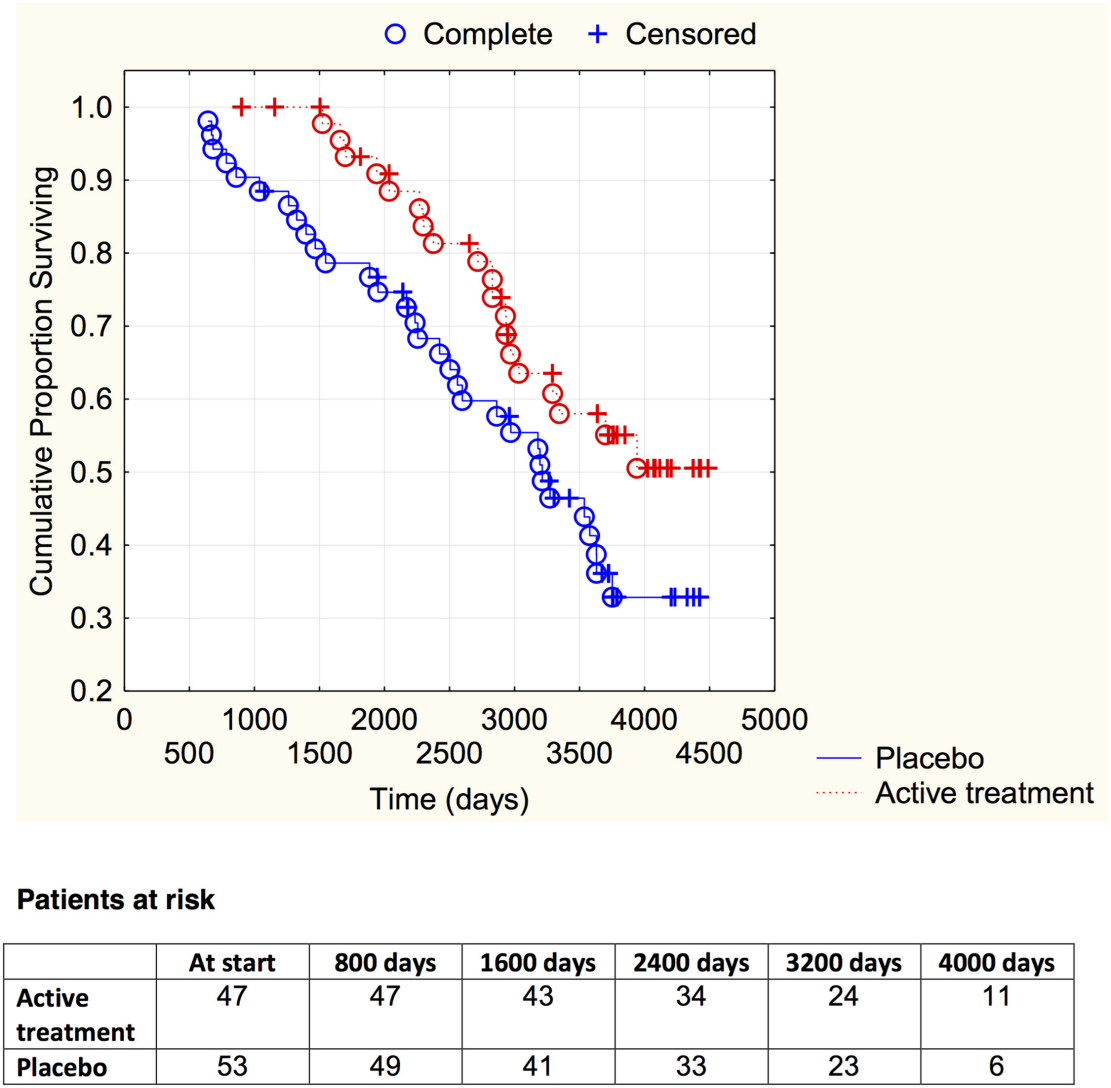
Kaplan-Meier graph illustrating cardiovascular mortality in the study population with ischemic heart disease, in those supplemented with selenium and coenzyme Q10 versus placebo during four years on top of regular pharmaceutic treatment during a follow-up period of 10 years.

Analyzing those with a history of IHD, a trend towards less CV mortality could be seen in those on active treatment (19/47 vs. 31/53; *χ* ^2^: 3.25; *P* = 0.071). The CV mortality over time is demonstrated in a Kaplan-Meier graph (Fig 2).

However, applying this small study sample into a multivariate model revealed a significantly reduced risk of CV mortality for those on active treatment (HR: 0.51; 95%CI 0.27–0.97; *P* = 0.04) (Table 3).

**Table 3:** Cox proportional hazard regression analysis evaluating risk of cardiovascular mortality by supplementation of selenium and coenzyme Q10 combined in a multivariate model after 10 years of follow-up after 4 years of intervention to an elderly with ischemic heart disease from a community.

Variables	Hazard ratio	95%CI	*P*-value
Age	1.15	1.05–1.26	0.004
Smoking	2.50	1.10–5.70	0.03
Hypertension	1.79	0.84–3.82	0.13
Diabetes	1.77	0.93–3.36	0.08
NYHA class 3	1.73	0.89–3.34	0.10
Hb<120g/L	0.96	0.46–2.00	0.92
EF<40%	0.92	0.44–1.93	0.82
NT-proBNP Q4	0.72	0.36–1.42	0.34
Active treatment	0.51	0.27–0.97	0.04

Notes: EF: Ejection fraction; IHD: Ischemic heart disease; NT-proBNP: N-terminal fragment of proBNP; NYHA: New York Heart Association functional class; Q4: 4^th^ quartile

**Fig 3:**
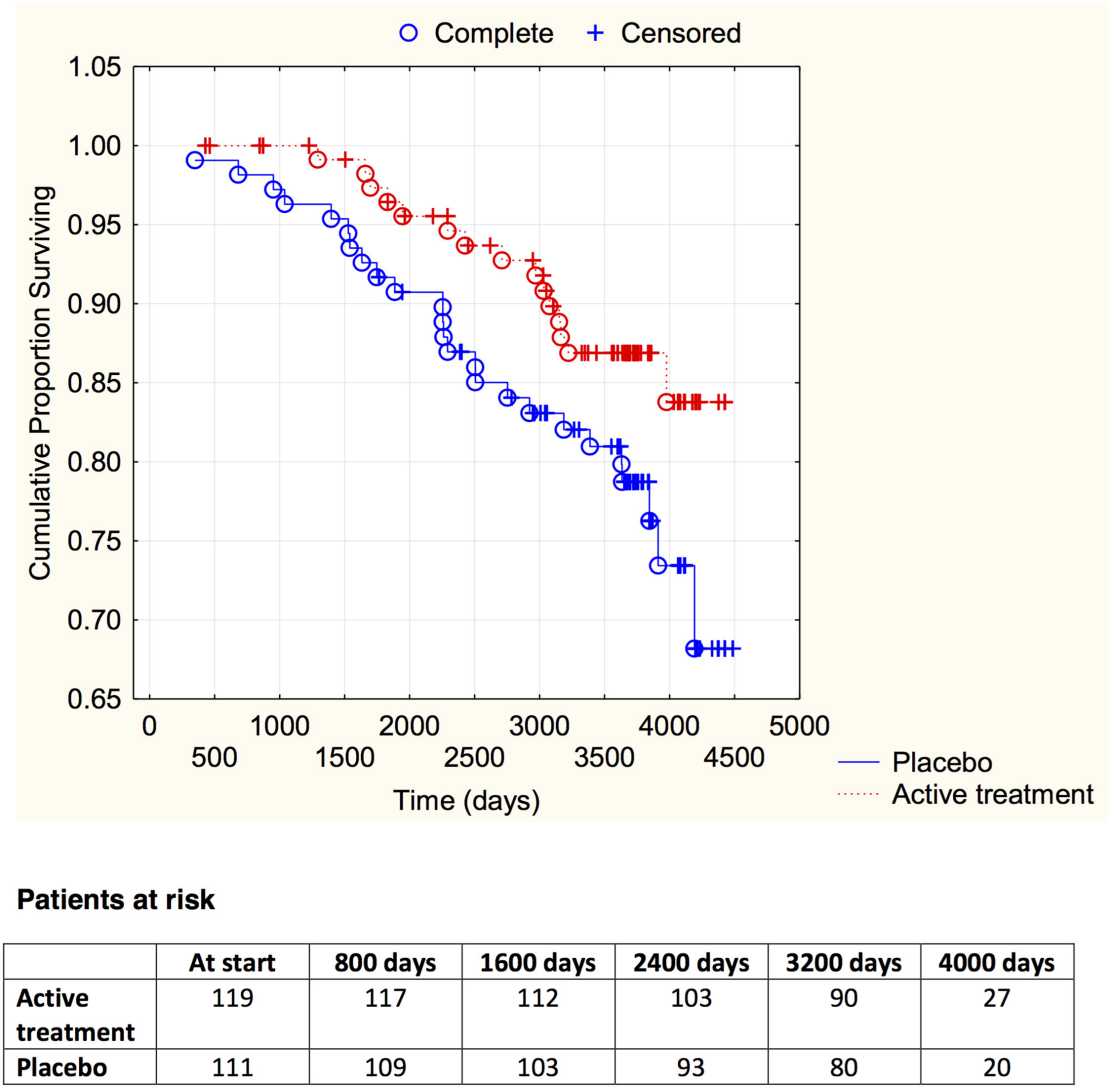
Kaplan-Meier graph illustrating cardiovascular mortality in the study population in NYHA functional class I, in those supplemented with selenium and coenzyme Q10 versus placebo during four years on top of regular pharmaceutic treatment during a follow-up period of 10 years.

**Fig 4:**
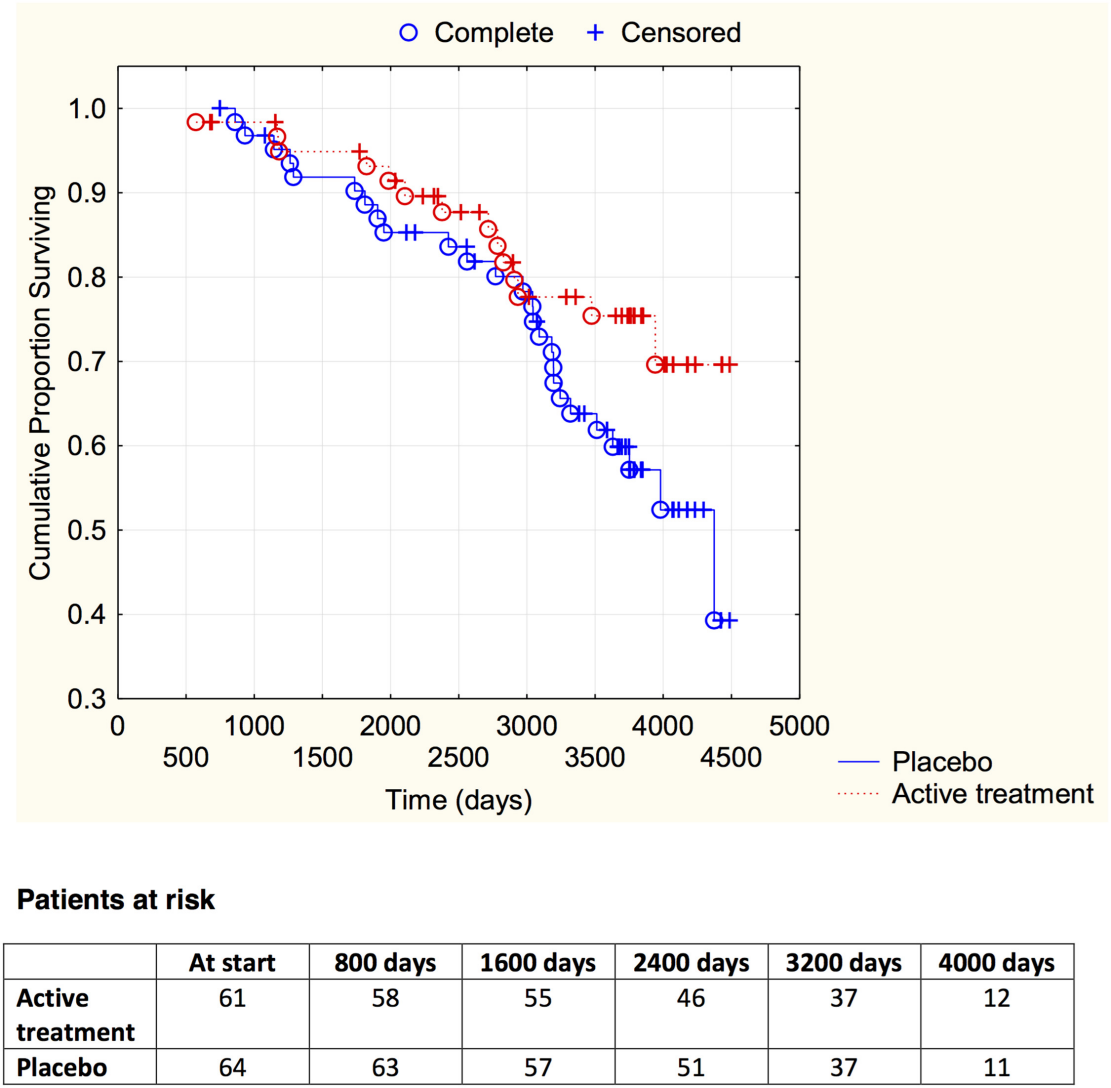
Kaplan-Meier graph illustrating cardiovascular mortality in the study population in NYHA functional class II, in those supplemented with selenium and coenzyme Q10 versus placebo during four years on top of regular pharmaceutic treatment during a follow-up period of 10 years.

**Fig 5:**
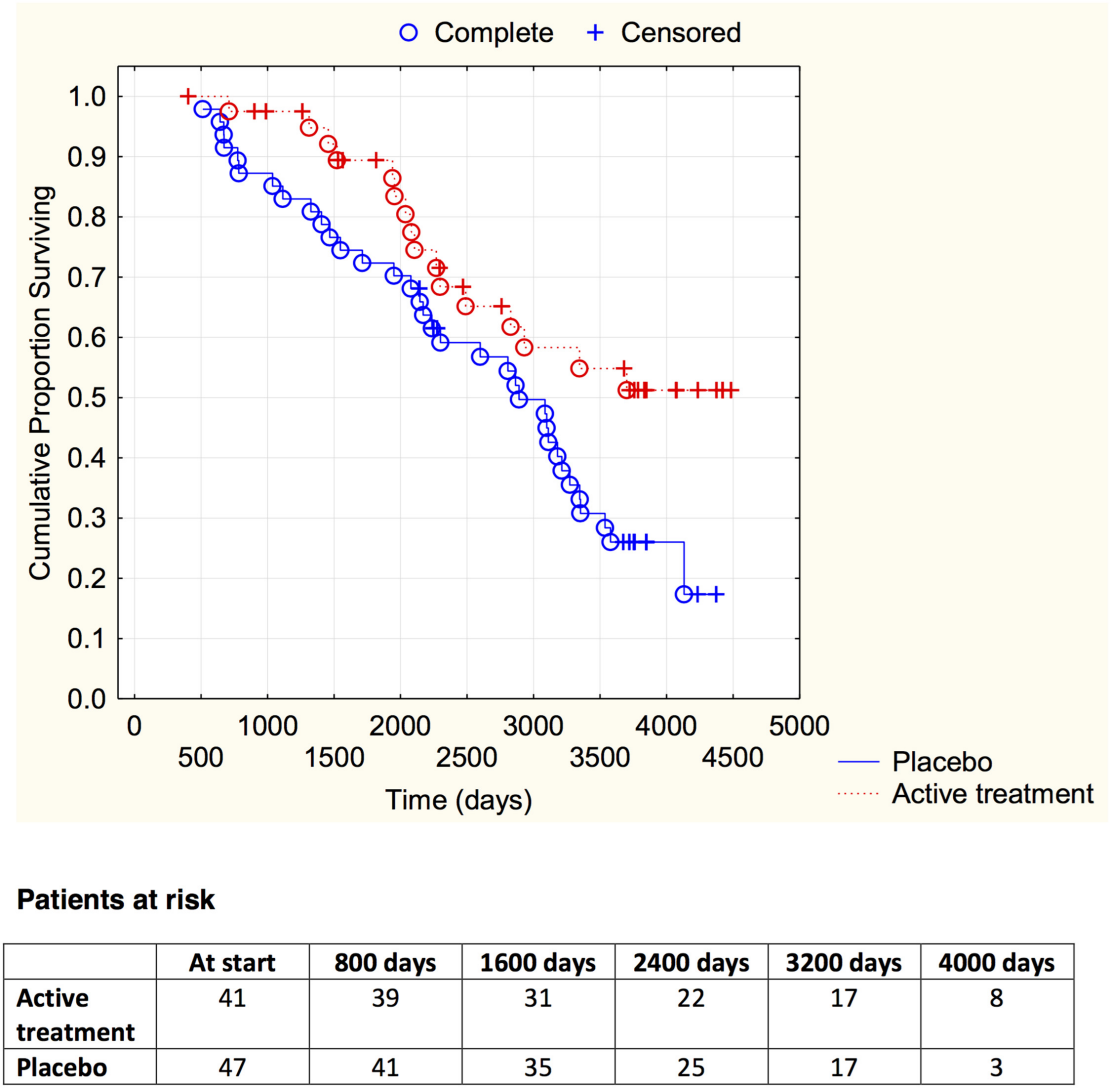
Kaplan-Meier graph illustrating cardiovascular mortality in the study population in NYHA functional class III, in those supplemented with selenium and coenzyme Q10 versus placebo during four years on top of regular pharmaceutic treatment during a follow-up period of 10 years.

Finally, by evaluating participants with different functional class according to the NYHA classification, it could be demonstrated that irrespective of the functional class, those on active treatment had less CV mortality (NYHA I: active treatment: 19/119 vs. placebo: 26/111; *χ* ^2^: 3.83; *P* = 0.05; NYHA II: active treatment: 14/61 vs. placebo: 26/64; *χ* ^2^: 4.48; *P* = 0.03; NYHA III; active treatment: 16/41 vs. placebo: 34/47; *χ* ^2^: 9.91; *P* = 0.002). The distribution of CV mortality over time in the different NYHA classes is presented in Figs 3–5.

### Other mortality within 10 years

A reduced all-cause mortality in the active group compared to the placebo group (98/221 vs. 120/222; *χ* ^2^:4.18; *P* = 0.041) could also be seen. Evaluating mortality in malignancies was performed, with no differences between the two groups (active treatment: 20/221 vs. placebo 19/222; *χ* ^2^:0.03; *P* = 0.86).

## Discussion

The results presented in this study are from a 10-year follow-up of a project in which selenium and coenzyme Q10 were given as a combined dietary supplement to 443 elderly community inhabitants. The present data indicate a post intervention persistency of the protection against CV mortality. The mortality rate at 5.2 years of follow-up, and at 10 years of follow-up are presented in Table 4. In the table the figures from official Swedish mortality statistics are also added in comparison. However, as the sample size of the present study is limited, the figures should be interpreted with caution.

**Table 4:** Mortality rate in the active treatment group compared to the placebo group, and to official mortality statistics.

	5.2 years of follow-up		10 years of follow-up	
	All-cause mort rate	Cardiovasc mort rate	All-cause mort rate	Cardiovasc mort rate
Active group	2433	1130	4427	2079
Placebo group	3115	2423	5400	3870
Reference pop	5794	2144	15,241	6998

Note: Mortality rate expressed as mortality /100,000/year

Note: Reference group: Official Swedish mortality statistics based on the age group 80–84 in the 5.2 year follow-up, and on the age group 85 years and above in the 10 years follow-up.

Surprisingly, the 48-month intervention appears to be accompanied by durable cardiovascular protection after termination of the supplements. From the analyses of subgroups it could be verified that patients with IHD, and/ or diabetes had an increased cardiovascular risk, but they appeared to be equally protected by the active combined supplementation treatment. And irrespective of the functional class of the participants, positive effects of the intervention could be seen. However, as the subgroups were small, the overall interpretation of our results is difficult.

Positive effects could be observed in males as well as in females, although a trend towards a better protective effect in females might be suggested. A recent German study of healthy elderly also suggested gender differences [25] as it reported lower levels of coenzyme Q10 and ubiquinol in females.

Increased oxidative stress[26–28], and inflammatory response[29–31], which may contribute to the progression of atherosclerosis, have been reported in elderly people.

It is well known that both selenium and coenzyme Q10 are needed for defense mechanisms against oxidative stress, a condition that may accompany any inflammation, and thus continuously affect any human cell throughout its lifetime. Endothelial cells and platelets in elderly people may be particularly exposed as they surround the continuous oxygen transport in the circulation, accompanied by increasing inflammatory response with age [32, 33]. It has been shown that increased oxidative stress is associated with impaired cardiac ventricular function. This can occur in the absence of myocardial infarction [34], although it is more obvious after an infarction[35]. Selenium, through selenium-containing enzymes, is a strong antioxidant, and optimal levels are needed for the maintenance of normal cell function. Ubiquinone is also essential for optimal antioxidant function. Positive effects of supplementation of coenzyme Q10 on endothelial function have also been reported in the literature [36].

An important question of the present evaluation was related to the possible persistency of the effect of supplementation on cardiovascular mortality after termination of the supplementation. From Table 1 it could be noted that from the variables EF<40%, and NYHA class III, thus those most diseased from a cardiovascular perspective, a more pronounced reduction of the amount of participants still alive could be seen in the placebo group between end of intervention and at the12 years of follow-up, compared to the active treatment group. This might indicate that the part most diseased died to a greater extent in the placebo group compared to the active treatment group also during the time after the termination of the intervention. An explanation might be a decrease of the progression of the cardiovascular disease during the intervention as could be seen from increased inflammatory activity that is reduced by the intervention [37] and where at the end result, if not given intervention, increased atherosclerosis and macroscopic cardiovascular injuries as a result could be registered up to 6 years after the termination of the intervention.

As the intervention was unusually long—four years—the effect of supplementation of selenium and coenzyme Q10 in this population, with obvious suboptimal plasma levels of selenium, is probably more profound than in individuals with marginally lowered selenium. The mean plasma selenium in the present population was about 67 μg/L [15], whereas optimization of the extracellular selenoprotein P requires plasma levels of 120 μg/L [38]. Here, it is relevant that selenium-containing enzymes operate not only as anti-oxidants, but also as anti-inflammatory and anti-atherothrombotic agents [39–41]. Thus, Helmersson et al. in a 27-year follow-up of Swedish males with a mean selenium concentration of 77 μg/L [42, 43] showed that high selenium concentration predicted lower signs of oxidative stress and reduced signs of inflammation, as seen inter alia from PGF_2α_. It should be noted that Swedish selenium levels are much lower than those reported from US populations [43]. The elderly are also more likely to have low levels of coenzyme Q10. Furthermore, a complex relationship between coenzyme Q10 and selenium exists, in which selenium is needed in order to facilitate reduction of coenzyme Q10 to ubiquinol, the active form. [16, 44]. A hypothesis that can be proposed from our results is that the intervention with selenium combined with coenzyme Q10 inhibits the pathogenesis of irreversible, presumably structural, changes preceding cardiovascular events.

However, part of the results could also be explained by the fact that the community under study developed an increased interest in supplementation with selenium and coenzyme Q10, as previously mentioned, and continued the supplementation. However, if this were so, individuals randomized to placebo might also have started supplementation on their own.

Increased or decreased risk of malignancies has been discussed in the literature as a result of supplementation with selenium. In this report, no sign of increased or decreased malignancies could be seen in the intervention group. However, it should be admitted that the study sample was small, and not intended for detection of such associations.

That supplementation with antioxidants could have positive effects also after the intervention has terminated has been reported by Bonelli et al. [45]. They evaluated patients with recurrent adenomas and where a supplementation of vitamin A, C and E combined with 200microgram selenium was given during 5 years. In a 15 years cumulative follow-up they could present data indicating a persisting effect of the reduction on recurrences of adenomas in the active intervention group. The study is small with several limitations. However, taken together with the results presented in the present study this could support the explanation that an anti-oxidative effect during the intervention might influence the macroscopic consequences of the disease evaluated.

## Limitations

The sample size of this intervention is an important limitation, and this makes the evaluation difficult, and a greater sample would certainly have been better. However, as the baseline levels of both selenium and coenzyme Q10 were measured, and documented as low, the need for supplementation was documented. The choice of outcome variable was chosen as a defined and documented outcome variable, even if uncertainties also exist in death certificates.

As evaluations of different subgroups were performed, the small sample sizes become even more obvious, and the results should be interpreted with caution. However, we consider that the presented data, irrespective of interpretations, should be regarded as hypothesis-generating.

The limited age span also represents a limitation, making extrapolation of the results difficult. However, in an elderly population the incidence of comorbidities is higher compared to younger persons, and an effect on cardiovascular disease competes with other disease states, making the results even more intriguing.

No measurements of the serum levels of selenium, or coenzyme Q10 have been done during the follow-up period. However, as earlier stated, the probability is high that those who started supplementation on their own are equally distributed between the two groups. Secondly, the effect of those taking selenium and coenzyme Q10 during the follow-up period will result in diminishing the difference between the two evaluated groups, why an underestimation of the actual effect could be the result.

Finally the population was a homogenous white group, living in Sweden, making extrapolation of results to other groups with other selenium concentrations difficult.

## Conclusion

A 10-year evaluation of CV mortality in an elderly Swedish healthy population that was given dietary supplementation with selenium and coenzyme Q10 over a period of four years, indicated a reduced risk of cardiovascular mortality by 50% and a post-intervention persistency of protection against CV mortality. The result could also be seen in the different genders, in those with diabetes, and in those with IHD. However, the mechanism explaining the persistent effect remains to be elucidated. Since this was a small study, the observations should be regarded as hypothesis-generating.

## Supporting Information

S1 Fig CONSORT flow chart of the study. (DOC)

S1 Study Protocol See Appendix. (DOCX)
