# Supplementary material for: Reduced Cardiovascular Mortality 10 Years after Supplementation with Selenium and Coenzyme Q10 for Four Years: Follow-Up Results of a Prospective Randomized Double-Blind Placebo-Controlled Trial in Elderly Citizens
Source: PLoS One. 2015 Dec 1;10(12):e0141641. doi: 10.1371/journal.pone.0141641 (PMC4666408; doi:10.1371/journal.pone.0141641)
Supplement: S1 Fig — (DOC) [file pone.0141641.s001.doc]

**
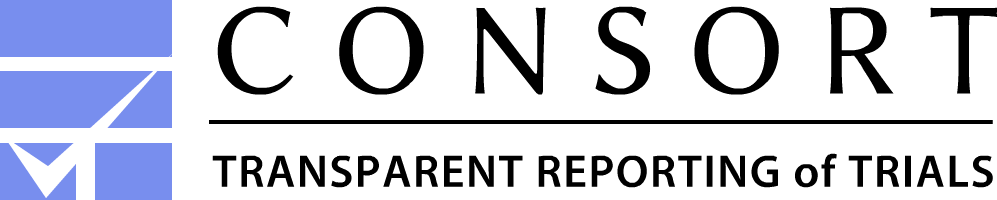
**

**CONSORT 2010 Flow Diagram**

**Allocation**

**Analysis**

**Follow-Up**

**Enrollment**

Assessed for eligibility (n=675)

Excluded (n=174 )

♦  Not meeting inclusion criteria (n= 0 )

♦  Declined to participate due to too many tablets (n=174 )

♦  Other reasons (seriously diseased) (n=58 )

Analysed (n=124 )
♦ Excluded from analysis (death n=28, drop outs n=69) (n= 97 )

Lost to follow-up (give reasons) (n= 97 )

Discontinued intervention (give reasons) (n= )

Allocated to intervention (n=221 )

♦ Received allocated intervention (n= 221 )

♦ Did not receive allocated intervention (give reasons) (n=0 )

Lost to follow-up (give reasons) (n=118 )

Discontinued intervention (give reasons) (n= )

Allocated to intervention (n=222 )

♦ Received allocated intervention (n= 222 )

♦ Did not receive allocated intervention (give reasons) (n= 0)

Analysed (n=104 )
♦ Excluded from analysis (death n=36, drop outs n=82) (n=118 )

Randomized (n= 443 )
